# Allergic reactions due to cow’s milk (CM) doses and triggering factors in 40 CM anaphylactic children during maintenance phase of specific oral tolerance induction (SOTI) treatment to CM in our unit

**DOI:** 10.1186/2045-7022-1-S1-O47

**Published:** 2011-08-12

**Authors:** Marta Vazquez, Jaime Lozano, Rosa Jimenez, Montserrat Alvaro, Olga Dominguez, Ana Maria Plaza

**Affiliations:** 1 Hospital Sant Joan de Deu, Pediatric Allergy and Clinical Immunology Department, Barcelona, Spain

## Background

Allergic reactions to CM doses generate controversy about SOTI treatment. Safety data during maintenance period of SOTI are limited.

## Aim

Describe allergic reactions to CM and trigger factors in CM anaphylactic patients during maintenance phase of SOTI (after achieving the maximum tolerated CM dose).

## Patients

40 CM anaphylactic children who underwent SOTI to CM in our Unit between 2006 and 2009.

## Methods

Interview with parents. Descriptive data analysis.

## Results

Median age: 10.6 years. Median maintenance period: 1.5 years. CM-IgE before SOTI: 101 KU/L. Median threshold dose at oral food challenge before SOTI: 2.5 ml. Median current daily CM dose: 200 ml.

Allergic reactions to CM: 29 children have had reactions, as follows:

A) Non anaphylactic reactions: 15 children. 8 children suffer daily from mild symptoms (4, mouth itching; 1, abdominal pain; 1, hives; 2, rhinitis) and 4 children from mild asthma 2-3/week, with no triggers associated. 6 children had 1 to 6 mild respiratory or digestive symptoms related to trigger factors.

B) 20 children have suffered from anaphylactic reactions, 50% of which are related to trigger factors. 14 patients had skin and respiratory involvement (rank: 65 to 1 episodes). 3 children had skin and digestive symptoms (rank: 4 to 1 episodes). 3 children, respiratory and digestive (rank:13 to 1 episodes). 7 children received epinephrine (39 doses). Number of children reacting to CM because of the following trigger factors: fasting, 5; exercise, 8 (66 episodes); lying, 12 (16 episodes); tiredness, 3; stress, 3; asthma exacerbation, 4; infection, 6. 9 children reacted to goat’s or sheep’s cheese and 4 to cow's cheese. 7 children have reduced in 10 to 75% their daily milk doses because of allergic reactions.

2 children withdrew SOTI during the maintenance period due to intense and frequent reactions.

## Conclusions

Allergic reactions are frequent in CM anaphylactic children during maintenance phase of SOTI. Exercise, asthma, infections, fasting, laying and stress are triggering factors. Strict follow up and education about allergic symptoms, triggers, dose reduction and treatment are crucial to contribute to safety of SOTI.

